# Production of Shelf‐Stable Fermented Maize Starch Flour for Akpan: Effects of Fermentation and Drying Conditions on Flour Characteristics and Final Product Quality

**DOI:** 10.1002/fsn3.72285

**Published:** 2026-09-17

**Authors:** Laurent Adinsi, Fernande G. Honfo, Agnes Mehoba

**Affiliations:** ^1^ Plateau Department National University of Agriculture Ketou Benin; ^2^ Faculty of Agronomics Sciences University of Abomey‐Calavi Cotonou Benin

**Keywords:** acidity, optimization, syneresis, texture, yogurt

## Abstract

Akpan is a Beninese yogurt‐like product whose quality and usability depend on fermented maize starch (FMS), a traditionally produced substrate characterized by inconsistent quality and limited shelf life. This study aimed to optimize the fermentation and drying conditions of maize starch to produce a stable FMS flour capable of yielding akpan with properties comparable to commercial akpan. A Box–Behnken response surface design was used to assess the effects of fermentation duration (24–72 h), drying temperature (45°C–65°C), and drying duration (4–6 h) on physicochemical and pasting characteristics of the flour, as well as the textural properties of akpan. Two commercial akpan samples were analyzed as benchmarks. Fermentation duration primarily influenced viscosity, while drying temperature significantly affected starch retrogradation and syneresis. Titratable acidity increased with both fermentation duration and drying temperature, while pH and lactic acid content remained relatively stable. Optimal conditions (60–72 h fermentation and ~55°C drying) produced FMS flour yielding akpan with titratable acidity (0.25–0.34 g/100 g), firmness (~14 g), and syneresis (< 18%), comparable to commercial products. Notably, pasting properties of FMS flour did not predict akpan viscosity, highlighting the importance of partial cooking conditions. These findings provide a basis for standardizing akpan production and improving its scalability and market potential.

## Graphical abstract

Optimization of fermented maize starch production through controlled fermentation and drying (60–72 h fermentation; ~55°C drying) yields a stable flour capable of producing akpan with quality attributes comparable to commercial products.

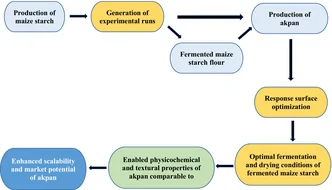

## 1 Introduction

Traditional cereal‐based foods fermented by lactic acid bacteria (LAB) are widely produced and marketed as street foods in both rural and urban areas of Africa due to their relative affordability (Osman et al. 2025). These products play a critical role in enhancing food security by providing accessible nutrition to diverse population groups, including resource‐constrained communities, while also contributing to dietary diversification. Fermentation has been demonstrated to be the most effective method for improving the nutritional and sensory qualities, as well as the shelf life, of cereal‐based foods compared to their unfermented counterparts (Dimoso et al. 2025). Consequently, such foods hold significant potential for integration into nutrition‐focused interventions targeting vulnerable populations, including children, women of reproductive age, and the elderly, thereby supporting the attainment of the United Nations Sustainable Development Goal 3 (SDG 3), which promotes good health and well‐being (Tanumihardjo et al. 2020). However, the predominantly spontaneous nature of traditional fermentation processes, coupled with inadequate control of processing conditions, increases the risk of contamination by undesirable microorganisms (Adesulu‐Dahunsi et al. 2020). This poses serious public health concerns, as contaminated products may be associated with diseases of varying severity, including diarrheal illnesses, carcinogenic effects, and, in extreme cases, mortality (Ademola et al. 2021).

In Benin, one such product is akpan, a yogurt‐like fermented food that is widely consumed and highly appreciated. It is typically produced through the partial cooking of fermented maize starch (FMS) and is commonly consumed with added ingredients such as sugar, sweetened milk, and ice (Akissoé et al. 2015). Owing to its demonstrated prebiotic effects on gastrointestinal health, as reported by Gullón et al. (2015), akpan is increasingly regarded as a potential functional food. However, FMS is predominantly produced through traditional, small‐scale processing methods, resulting in considerable variability in quality and a relatively short shelf life. These limitations constrain the consistency, scalability, and overall availability of akpan. Furthermore, during storage and distribution, akpan is subject to continuous acidification, sometimes accompanied by an increase in LAB and mold populations, and a reduction in viscosity (Akissoé et al. 2015). These changes highlight the product's short shelf life and underscore the limited production and distribution capacity of traditional processing systems. Recent findings by Sanya et al. (2024) emphasize the importance of standardizing FMS process, as this would ultimately enhance the quality and stability of akpan. Moreover, such standardization could improve the accessibility of FMS thereby enabling consumers to prepare akpan at any time according to their preferences.

One promising approach to addressing these challenges is the drying of FMS, which can be stored for extended periods and later reconstituted into akpan when needed. This strategy enhances shelf stability, minimizes post‐production losses, and improves convenience while also providing a foundation for modernizing traditional akpan processing to achieve greater consistency, scalability, and marketability, particularly in response to increasing urban demand. In this context, oven drying emerges as a viable and attractive preservation method for FMS, with potential to maintain product quality and functionality when appropriate drying parameters are applied. However, LAB fermentation can modify the composition and acidity of cereal substrates and consequently influence the formation of Maillard reaction products during subsequent thermal processing; therefore, drying conditions should be carefully controlled to limit undesirable heat‐induced reactions (Sahin et al. 2024). Similarly, Ertop and Coşkun (2018) demonstrated that extended oven drying of sourdough increases color intensity and browning, significantly influencing the overall consumer acceptance. Therefore, this study aimed to optimize the fermentation and oven‐drying conditions of maize starch slurry (MSS) to produce fermented maize starch flour capable of yielding akpan with physicochemical and textural attributes comparable to those of commercially produced akpan, while enhancing the flour's stability and process standardization.

## 2 Material and Methods

### 2.1 Experimental Design

A Box–Behnken response surface methodology (RSM) set by Statistica software was employed to assess the effects of MSS fermentation and drying conditions on the quality attributes of akpan. Three independent factors were investigated: MSS fermentation duration (24–72 h), drying temperature (45°C–65°C), and drying duration (4–6 h). The minimum and maximum levels of fermentation duration were defined based on a previous study (Sacca et al. 2012), while the drying temperature range was selected to prevent maize starch gelatinization (Coral et al. 2009), and the drying duration was established through preliminary experiments to produce FMS flour with a target water activity of approximately 0.6 (Anihouvi et al. 2006). The samples were dried in an oven and milled using a hammer mill (Lister‐DMA) to produce flour. The experimental design consisted of 15 trials, including three replicates at the central point (CP), as summarized in Table 1.

**TABLE 1:** Experimental matrix.

Experiments	Fermentation duration (h)	Drying duration (h)	Drying temperature (°C)
1	24	4	55
2	72	4	55
3	24	8	55
4	72	8	55
5	24	6	55
6	72	6	45
7	24	6	65
8	72	6	65
9	48	4	45
10	48	8	45
11	48	4	65
12	48	8	65
13©	48	6	55
14©	48	6	55
15©	48	6	55

Abbreviation: ©, Central point.

### 2.2 Akpan Production

White maize ( *Zea mays* L.) was purchased from Dantokpa Market in southern Benin and used to produce MSS. The whole maize kernels were boiled for 10 min, steeped for 15 h, wet‐milled, and wet‐sieved using tap water at a water‐to‐ground‐maize ratio of 5:1, following the method described by Bouniol et al. (2021). A preliminary test of akpan preparation was conducted with six skilled women processors familiar with producing akpan of desirable texture. Each processor was provided with 250 g of FMS flour obtained from the central point experiment, along with water and a gas cooker. The processors were allowed to independently determine the partial cooking conditions, including temperature, timing and necessity of water addition, and the quantity of FMS flour incorporated. Process parameters, including temperature, duration, water and FMS flour addition at each stage, were systematically recorded. This approach enabled the standardization of the akpan preparation procedure subsequently applied to the 15 FMS flour samples, as described below.

FMS flour (250 g) was divided into two equal portions (125 g each). The first portion was diluted with water to obtain a slurry containing 15% dry matter (approximately a 1:5 flour‐to‐water ratio), then heated in a water bath at 100°C until reaching 72°C–75°C, and subsequently cooled to 55°C at room temperature (28°C–30°C). The second portion was mixed with water to obtain a slurry containing 22.5% dry matter (approximately a 1:3 flour‐to‐water ratio) and was added, off heat, to the first slurry at 55°C. The resulting mixture was then allowed to cool at room temperature (28°C–30°C) and thus Akpan, a partially cooked product, was obtained.

Two commercial akpan samples were collected from independent processors operating at Abomey‐Calavi Market in southern Benin for comparison.

### 2.3 Physicochemical and Textural Characterization

#### 2.3.1 Dry Matter and Water Activity

Dry matter content (DMC) was determined on FMS flour and akpan samples by oven drying at 105°C to constant weight. Water activity was determined on FMS flour with a thermo‐hygrometer recorder C056696 (Rotronic HygroLab 2, Rotronic AG, Bassersdorf, Switzerland) (Anihouvi et al. 2006).

#### 2.3.2 Color

Color parameters were determined on FMS and FMS flour samples using a CR 410 chromameter (Konica Minolta Sensing, Sakai, Osaka, Japan) calibrated with a reference white ceramic whose color coordinates are: *Y* = 86.10; *x* = 0.3194 and *y* = 0.3369. The L*, a*, b* values were recorded, where L* corresponds to the luminance; a* the saturation in red and b* the saturation in yellow. The whiteness index was calculated as described by Rhim et al. (1999).

#### 2.3.3 pH and Titratable Acidity

pH was determined using a digital pH meter (inoLab pH 730, WTW GmbH, Weilheim, Germany) calibrated with buffers at pH 4.0 and 7.0 (WTW GmbH, Weilheim, Germany) on FMS, FMS flour and akpan samples, and titratable acidity, expressed as lactic acid equivalent, was measured by titrating 10 g using 0.1 N NaOH (Merck).

#### 2.3.4 Lactic Acid

Lactic acid was carried out on FMS flour samples by HPLC analysis using an Aminex HPX 87H column (300 × 7.8 mm; Bio‐Rad Laboratories Inc., Hercules, CA, USA) (Mestres and Rouau 1997). Extraction was performed on 50 mg of sample with 1 mL of 5 mM H_2_SO_4_ solution. The suspension was dispersed vigorously, then gently shaken for 1 h and centrifuged at 10,000 × *g* for 5 min. The supernatant was filtered through a 0.45 μm pore size filter and injected onto the HPLC system. The extraction and HPLC were performed in duplicate. Lactic acid content was detected according to its retention time and absorbance at 210 nm using a UV detector.

#### 2.3.5 Pasting Properties

There were measured on the FMS flours using a HAAKE Viscotester iQ Air rheometer (Thermo Fisher Scientific, Karlsruhe, Germany) as described by Bouniol et al. (2021). Three parameters expressed in Poise (*p*) were collected: peak viscosity, final viscosity, and setback.

#### 2.3.6 Syneresis

Akpan (20 g) was placed in centrifuge tubes and centrifuged at 350 × g using a refrigerated centrifuge (VWR MEGA STAR 600R, VWR International GmbH, Darmstadt, Germany) for 10 min at 4°C. The resulting supernatant was carefully collected and weighed, and the syneresis index was subsequently calculated. Syneresis (%) = 100 * weight of supernatant (g)/weight of akpan sample (g).

#### 2.3.7 Instant Apparent Viscosity

Instant apparent viscosity of akpan samples was measured using a HAAKE Viscotester iQ Air rheometer (Thermo Fisher Scientific, Karlsruhe, Germany). A 28 g portion of homogenized sample was heated to 35°C for 3 min under continuous stirring at 160 rpm (Akissoé et al. 2015), after which the final viscosity was recorded and expressed in Centipoise (cP).

#### 2.3.8 Texture

It was determined on akpan sample by penetration measurements using TA‐XT plus, Texture Analyzer (Stable Micro Systems, Godalming, UK) equipped with 5 kg load cell and cylindrical probe (20 mm of diameter). The instrument was adjusted to the following conditions: cylindrical probe, probe diameter: 20 mm; penetration speed: 1.0 mm/s; penetration distance, 20 mm into surface. The software used was Exponent (Stable Micro Systems, 2006, version 5.0). 80 mL of akpan sample was analyzed in each cup. Firmness (g) (maximum force, i.e., exerted on the sample), defined as the force necessary to attain a given deformation was collected.

### 2.4 Statistical Analysis

The experimental data collected from Box–Behnken design were analyzed by the response surface regression procedure using a second‐order polynomial equation. All these statistical analyses and the graphical presentations were performed using Statistica software (version 7.1, Stat Soft France 2006).

## 3 Results and Discussion

### 3.1 Changes in Dry Matter, Color and Acidity During MSS Fermentation

MSS fermentation (24–72 h) resulted in a significant reduction in dry matter content, decreasing from 35.2% to 31.9% (Table 2), likely due to the metabolic activity of fermentative microorganisms (Li et al. 2023). In contrast, no significant changes were observed in the whiteness index, pH, or titratable acidity (Table 2). The relative stability of pH and titratable acidity may be attributed, at least in part, to the buffering capacity of solubilized proteins within the maize matrix, as proteins can contribute substantially to the pH‐buffering capacity of food systems (Yu et al. 2024). Similarly, Sanya et al. (2024) reported pH values ranging from 3.1 to 4.2 during MSS fermentation (6–72 h), consistent with the findings of the present study, although higher titratable acidity values (0.7–1.5) were observed in their work.

**TABLE 2:** Physicochemical properties of fermented maize starch and fermented maize starch (FMS) flour as affected by fermentation and drying conditions.

Products	Dry matter (%)	a_w_	Whitness index	pH	Titratable acidity (g/100 g, db)	Lactic acid (g/100 g, db)
Fermented maize starch						
Fermented duration (h)						
24 (*n* = 4)	35.2^a,^ *	nd	92.99^a^	3.15^a^	0.29^a^	nd
48 (*n* = 7)	33.8^b^		92.98^a^	3.35^a^	0.24^a^	
72 (*n* = 4)	31.9^c^		92.99^a^	3.15^a^	0.26^a^	
Fermented maize starch flour						
Experiments						
1	90.1	0.62	88.4	3.00	0.13	0.95
2	94.6	0.50	86.5	3.14	0.37	1.08
3	95.4	0.44	85.5	3.42	0.17	0.90
4	92.0	0.54	88.1	3.37	0.21	1.07
5	91.4	0.56	85.9	3.82	0.11	0.35
6	91.7	0.57	88.8	3.36	0.22	0.97
7	95.6	0.47	88.5	3.43	0.16	0.95
8	94.2	0.54	85.1	3.09	0.32	1.55
9	90.7	0.57	88.3	3.55	0.11	0.25
10	92.0	0.53	83.5	3.37	0.18	0.93
11	92.4	0.59	85.8	3.25	0.26	1.31
12	95.2	0.43	84.9	3.17	0.25	1.29
13©	91.7	0.51	88.3	3.32	0.22	0.81
14©	94.2	0.47	90.3	3.41	0.17	0.93
15©	92.0	0.50	88.7	3.29	0.27	1.30
Model	Linear	Linear	Linear	Linear	Linear	Linear
*R* ^2^	0.85	0.91	0.82	0.42	0.87	0.71
Fermentation duration	0.53 (+)	0.79 (−)	0.37 (+)	0.34 (−)	**0.006** (+)	0.10 (+)
Drying temperature	**0.02** (+)**	**0.02** (−)	0.99 (−)	0.31 (−)	**0.03** (+)	0.05 (+)
Drying duration	0.09 (+)	**0.006** (−)	0.12 (−)	0.62 (+)	0.67 (−)	0.53 (+)

Abbreviations: (+), positive effect of factor on dependent variable; (−), effect of factor on dependent variable; ©, central point; db, dry basis; nd, not determined.

* Mean with identical letters indicate no significant difference at *p* < 0.05.

** Probabilities in bold indicated significant effect at *p* < 0.05.

### 3.2 Physicochemical Characteristics of FMS Flour

No significant effects of FMS flour processing conditions were observed on whiteness index, pH, and lactic acid content (Table 2). Although pH can serve as a rapid qualitative indicator of acidity development, its reliability is limited in systems with substantial buffering capacity, particularly due to soluble proteins in the slurry, which may account for the observations in the present study.

Lactic acid, a key metabolite of LAB fermentation, contributes to acidification and sourness in fermented cereals and may enhance microbial stability (Liu et al. 2026). Unexpectedly, lactic acid recognized as the predominant organic acid in FMS (Akissoé et al. 2015) was not significantly influenced by fermentation duration, in contrast to the findings of Sanya et al. (2024), nor by drying duration. This lack of variation may be attributed to a reduction in lactic acid content during the drying process, potentially masking the effects of fermentation duration. A similar trend was reported by Balli et al. (2023) for pearl millet, fermented for 72 h before drying. Significant reductions in organic acids, including lactic acid, were observed after spray‐drying, suggesting that drying losses may partly mask the effects of fermentation on lactic acid content in FMS. Therefore, distinguishing differences in lactic acid content among FMS flour samples would likely require milder drying conditions than those applied in this study, where the least severe treatment involved drying at 45°C for 4 h.

**FIGURE 1:**
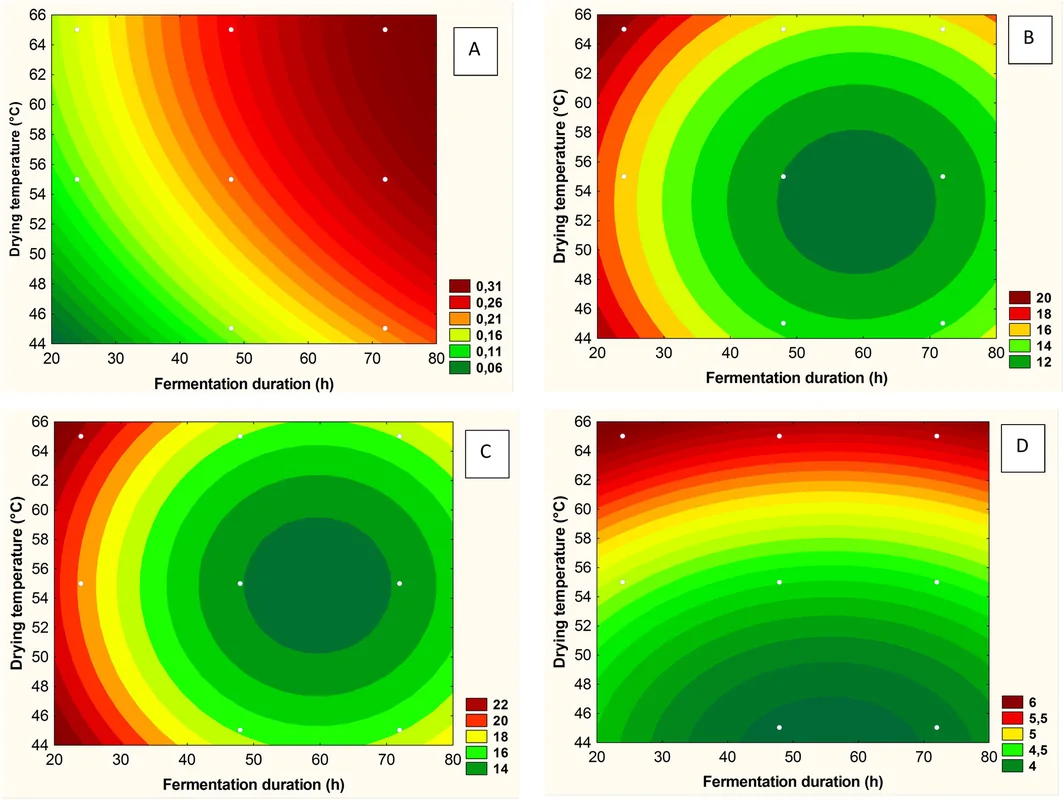
Response surface models for titratable acidity (A), peak viscosity (B), final viscosity (C), and setback (D) of the fermented maize starch flours.

Models describing the evolution of dry matter, water activity, and titratable acidity exhibited predominantly linear behavior (Table 2). Dry matter content of FMS flour increased significantly with increasing drying temperature, reaching values above 90%. This trend can be attributed to internal moisture migration followed by evaporation from the material surface into the surrounding air. Water activity showed an inverse relationship with both drying duration and temperature, increasing as these parameters decreased, with a maximum value of approximately 0.6. Given that dry matter content exceeded 90% and water activity remained below 0.6, FMS flour samples produced under the studied conditions are expected to be shelf‐stable at ambient temperature for several months, in agreement with previous works (Prada‐Ramírez et al. 2024). Therefore, under these conditions, neither dry matter nor water activity constitutes a critical constraint for FMS flour production. In contrast, titratable acidity was significantly influenced by both fermentation duration and drying temperature, which independently contributed to its increase (Table 2). The observed rise in titratable acidity following drying may be associated with secondary microbial metabolic activity. Such processes have been reported to promote the accumulation of organic acids, particularly lactic, acetic, and butyric acids (Kitessa 2024). Based on the response surface contour plot (Figure 1A), the critical fermentation duration and drying temperature were identified as approximately 48 h and 55°C, respectively. Below these thresholds, titratable acidity remained below 0.25 g/100 g, a level considered critical when compared with typical values reported for FMS samples.

### 3.3 Pasting Properties of FMS Flour as Affected by Fermentation and Drying Conditions

The pasting parameters were adequately described by linear and/or quadratic models (*R* ^2^ > 0.8), indicating a good fit to the experimental data, with fermentation duration and drying temperature identified as the main influencing factors (Table 3). The predicted responses are presented as response surface contour plots (Figure 1B–D).

**TABLE 3:** Pasting properties of fermented maize starch (FMS) flour as affected by fermentation and drying conditions.

Experiments	Peak viscosity (*p*)	Final viscosity (*p*)	Setback (*p*)
1	18.2	22.5	4.3
2	10.6	11.7	4.1
3	15.8	16.7	4.5
4	11.9	15.7	4.7
5	16.0	18.1	4.9
6	11.2	14.2	3.3
7	17.4	22.0	6.0
8	17.6	17.9	6.0
9	15.3	18.8	5.2
10	13.1	15.8	2.7
11	11.8	13.4	5.0
12	17.0	17.7	5.6
13©	9.7	13.2	4.5
14©	9.7	10.9	3.7
15©	13.3	15.4	4.6
*R* ^2^	0.9	0.89	0.84
Fermentation duration (L)	**0.005 (−)** *	**0.006 (−)**	0.48 (−)
Fermentation duration (Q)	**0.015 (−)**	**0.02 (−)**	0.46 (−)
Drying temperature (L)	0.75 (+)	**0.04 (−)**	**0.02 (+)**
Drying temperature (Q)	**0.013 (−)**	**0.02 (−)**	0.22 (−)
Drying duration (L)	0.67 (+)	0.91 (−)	0.52 (−)
Drying duration (Q)	0.76 (+)	0.77 (−)	0.67 (+)

Abbreviations: (+), positive effect of factor on dependent variable; (−), negative effect of factor on dependent variable; ©, central point; L, Linear effect of factor on dependent variable; Q, Quadratic effect of factor on dependent variable.

* Probabilities in bold indicated significant effect at *p* < 0.05.

The evolution of peak and final viscosities was best described by a model including a linear negative effect of fermentation duration (*p* = 0.005 and 0.006, respectively) and a quadratic negative effect of drying temperature (*p* = 0.01 and 0.02, respectively). Similar reductions in viscosity with increasing fermentation duration have been reported for fermented cereal flours (Sobowale et al. 2025). These changes may be associated with structural modifications of starch during fermentation, including alterations in amylopectin chain distribution, molecular organization, and crystalline order, which can affect granule swelling and pasting behavior (Zheng et al. 2025). In addition, high drying temperatures further limit swelling capacity, as reflected in decreased peak viscosity (Cheng et al. 2024). However, the effect of drying temperature on both viscosities was strongly dependent on fermentation duration (Figure 1B,C). Below 30 h of fermentation, the effect was negligible and viscosities remained high; beyond this threshold, a bipolar pattern emerged, with 55°C representing a critical point above or below which both viscosities increased. Peak viscosity is also influenced by starch granule size, which governs swelling behavior and pasting properties (Salcedo Salazar et al. 2026). Consistent with this, Bouniol et al. (2021) reported relatively high peak viscosities in local cassava varieties, corresponding with consumer and processor preferences for cassava‐based stiff dough. In the present study, FMS flour samples exhibited high peak and final viscosities at fermentation durations below 30 h, irrespective of drying temperature, indicating a critical threshold beyond which both parameters decline (Figure 1B,C).

Setback, a key functional characteristic of starchy products, reflects the gelling capacity of starch arising from the reassociation of leached amylose molecules (Bouniol et al. 2021). Along with final viscosity, setback provides an indication of the extent of amylose retrogradation during cooling and serves as a critical parameter for assessing food quality. In the present study, setback was best described by a positive linear effect of drying temperature (Table 3), consistent with observations by Timm et al. (2020) for maize starch dried between 30°C and 90°C. This increase may be attributed to enhanced starch retrogradation during cooling, potentially associated with increased amylose recrystallization and the formation of a stronger three‐dimensional gel network (Zhang et al. 2025). Saleh et al. (2020) reported that in starch‐containing yogurt systems, starch characteristics strongly influence gel firmness, viscosity, and syneresis during storage, indicating that the suitability of a high‐setback starch depends on the desired textural and storage properties of the final product. In the present study, setback values increased significantly above drying temperature of 55°C, irrespective of fermentation duration, suggesting this temperature as a critical threshold for modulating the gelling properties of FMS flour (Figure 1D).

The above observations highlighted a significant interaction between fermentation duration and drying temperature in FMS flour production: fermentation duration predominantly governs peak and final viscosities, while drying temperature primarily influences retrogradation behavior. A fermentation duration of 30 h combined with drying at approximately 55°C is expected to yield high viscosity while limiting retrogradation. Under these conditions, and consistent with Saleh et al. (2020) the resulting FMS flour should be suitable for the production of akpan, which requires starch stability under low‐temperature storage conditions.

### 3.4 Physicochemical and Textural Properties of Commercial Akpan

Akpan samples collected from the market exhibited a dry matter content of approximately 15% (Table 4). The products were characterized by an instant apparent viscosity of 113 cP and pronounced acidity, with a pH of 3.1 and a mean titratable acidity of 0.27 g/100 g (Table 4). Akissoé et al. (2015) observed similar titratable acidity (0.26 g/100 g) and dry matter content (14.1%), but reported a higher pH (3.6) and substantially greater instant apparent viscosity (6120 cP). This discrepancy in viscosity may be attributed to differences in analytical methodologies, particularly the use of distinct measurement techniques (e.g., rheometer versus rapid visco analyzer), which are known to significantly influence viscosity measurements. Commercial akpan displayed a firmness of approximately 14 g while following refrigeration, a degree of water separation was observed, with an average syneresis value around 16%. To the best of our knowledge, this study is the first to document both the firmness and syneresis of akpan. Syneresis represents a critical visual and quality attribute of yogurt‐like products, strongly influencing consumer perception and acceptance (Arab et al. 2023). Generally, lower syneresis values are associated with improved product stability and enhanced consumer acceptability.

**TABLE 4:** Physicochemical and textural properties of commercial akpan and experimental akpan as affected by fermentation and drying conditions of fermented maize starch.

Products	Number	Dry matter (%)	pH	Titratable acidity (g/100 g, db)	Syneresis (%)	Instant apparent viscosity (cP)	Firmness (g)
Commercial akpan	1	15.2	3.18	0.25	20.4	111.0	13.9
	2	14.6	3.08	0.29	12.1	116.2	14.3
Experiment akpan	1	18.0	3.45	0.18	17.6	62.5	13.6
	2	17.9	3.22	0.34	15.1	127.5	14.5
	3	17.6	3.46	0.21	14.7	97.1	13.8
	4	17.7	3.49	0.28	20.1	31.0	13.2
	5	18.0	4.00	0.10	20.4	82.1	13.5
	6	17.9	3.48	0.18	14.1	78.1	13.4
	7	17.5	3.51	0.17	25	132.2	13.9
	8	17.8	3.19	0.30	24	120.9	13.8
	9	17.8	3.70	0.14	14	60.8	13.3
	10	17.9	3.50	0.17	11.4	32.1	13.2
	11	17.9	3.39	0.23	22.5	35.4	13.2
	12	17.6	3.30	0.24	26	82.4	12.9
	13©	17.8	3.52	0.18	19.0	89.9	13.3
	14©	17.9	3.39	0.18	15	102.4	13.0
	15©	18.2	3.35	0.23	19.2	105.5	12.9
	*R* ^2^	0.62	0.55	0.9	0.83	0.84	0.88
	Fermentation duration (L)	0.86 (+)*	0.12 (−)	**0.003** (+)	0.98 (−)	0.92 (+)	0.87 (+)
	Fermentation duration (Q)	0.45 (+)	0.71 (−)	0.47 (−)	0.87 (−)	0.32 (−)	**0.005** (−)
	Drying temperature (L)	0.19 (−)	0.17 (−)	**0.01** (+)	**0.02** (+)	0.17 (+)	0.73 (+)
	Drying temperature (Q)	0.52 (+)	0.81 (−)	0.13 (+)	0.35 (−)	0.57 (+)	0.99 (+)
	Drying duration (L)	0.25 (−)	1 (+)	0.88 (+)	0.66 (+)	0.51 (−)	0.09 (−)
	Drying duration (Q)	0.4 (+)	0.56 (+)	0.06 (−)	0.38 (+)	0.06 (+)	0.42 (−)

Abbreviations: (+), positive effect of factor on dependent variable; (−), negative effect of factor on dependent variable; ©, central point; db, dry basis; L, Linear effect of factor on dependent variable; Q, Quadratic effect of factor on dependent variable.

* Probabilities in bold indicated significant effect at *p* < 0.05.

### 3.5 Physicochemical and Textural Properties of Akpan as Affected by Processing Conditions of FMS Flour

The preparation process of akpan, which involves the addition of water based on the dry matter content of FMS flour, resulted in Akpan with comparable dry matter contents (17.5%–18.2%), irrespective of the processing conditions of the FMS flour (Table 4). Notwithstanding this consistency, the akpan produced in this study exhibited a relatively higher dry matter content than that of commercial akpan. Similarly, the pH of the akpan appeared to be independent of the production conditions of the FMS flour, likely reflecting the same underlying factors previously discussed for the flour itself. This observation is supported by a strong and significant positive correlation (*r* = 0.86) between the pH values of the FMS flour and the corresponding akpan. Overall, the pH values of the akpan samples were substantially higher than those of commercial akpan, with differences ranging from 0.1 to 0.9 pH units. In addition to lactic acid, pH and titratable acidity provide complementary information on the acidification status of akpan. These parameters are relevant to the quality of fermented products because acidification can influence microbial stability and sensory attributes, particularly sourness (Hernández Figueroa et al. 2024; Ozabor et al. 2026). However, because microbial populations, sensory responses and nutritional composition were not directly measured in the present study, the effects of these parameters on these attributes cannot be established experimentally and should therefore be considered as potential quality implications.

**FIGURE 2:**
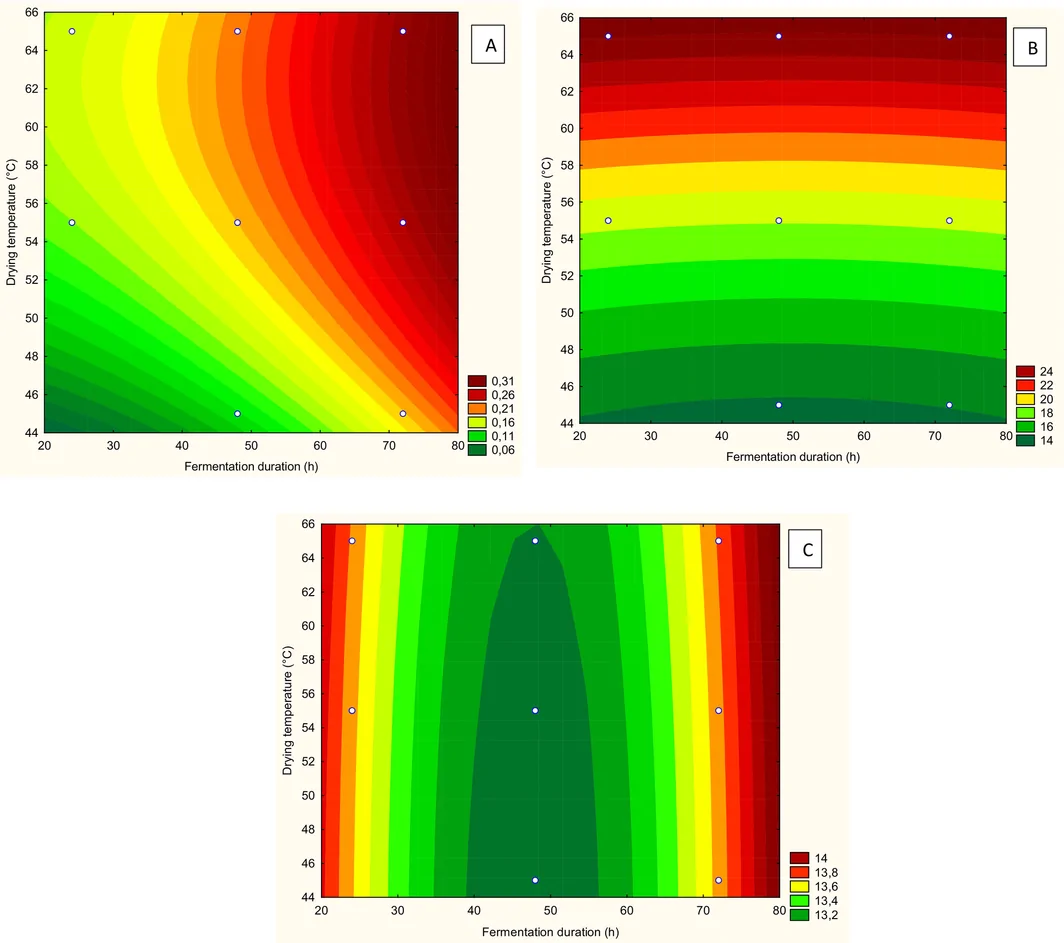
Response surface models for titratable acidity (A), syneresis (B), and firmness (C) of the akpan.

Titratable acidity was well described by positive linear relationships with both fermentation duration and drying temperature (Table 4), in agreement with trends previously observed for FMS flour. These results suggest that changes in titratable acidity during the cooking step were likely similar across all flour samples, thereby emphasizing the importance of standardizing the cooking process. As illustrated in Figure 2A, fermentation durations exceeding 60 h, combined with drying temperatures above 55°C, yielded FMS flour that, upon reconstitution, produced akpan with acidity levels comparable to those of commercial products. In contrast, Sacca et al. (2012) reported that FMS used in traditional akpan production is typically obtained after approximately 48 h of fermentation. The longer fermentation duration observed in the present study likely compensates for potential losses of lactic acid occurring during the drying process, as reported by Semenov et al. (2024).

In contrast to FMS flour, for which fermentation duration and drying temperature significantly influenced pasting properties, the instant apparent viscosity of akpan was not affected by these factors (Table 4). This observation indicates the absence of a direct relationship between the pasting characteristics of FMS flour and the viscosity of akpan. This is further supported by the non‐significant correlations between instant apparent viscosity of akpan and both final viscosity (*r* = 0.01) and peak viscosity (*r* = 0.15) of FMS flour. These findings suggest that pasting parameters of FMS flour, determined under standardized analytical conditions, are not reliable predictors of the flow behavior of akpan at the point of consumption. As reported by Kewuyemi et al. (2024), this discrepancy may be attributed to the influence of processing‐related variables during partial cooking such as water‐to‐flour ratio, mixing intensity, and temperature profile as well as post‐pasting structural transformations. Collectively, these factors exert a dominant effect on the instant apparent viscosity of akpan, thereby decoupling it from the intrinsic pasting properties of the FMS flour.

Akpan syneresis was adequately described by a linear model and was found to be predominantly influenced by drying temperature (Table 4), with values remaining within the range reported for commercial akpan. A strong positive correlation was observed between syneresis and setback values (*r* = 0.84), consistent with findings for yogurt‐type products (Arab et al. 2023). Notably, 55°C was identified as a critical drying temperature for FMS flour, beyond which a significant increase in akpan syneresis was observed (Figure 2B). Firmness, another key textural attribute, is generally regarded as desirable in yogurt‐like products when elevated. In this study, firmness was best described by a positive quadratic model as a function of fermentation duration. This observation aligns with the findings of Anwar et al. (2025), who reported that extended fermentation enhances yogurt firmness. Accordingly, akpan firmness reached maximal values at fermentation durations of approximately 72 h (Figure 2C).

### 3.6 Optimal Processing Conditions for FMS Flour to Achieve Commercial‐Quality Akpan

Overall, the results indicate that the optimal processing conditions for FMS flour intended for akpan production require a careful balance between fermentation duration and drying temperature to achieve physicochemical and textural characteristics of akpan comparable to those of commercial product. In particular, fermentation duration exceeding 60 h, combined with drying temperature around 55°C, appear to provide the most suitable compromise. Under these conditions, the resulting akpan exhibits titratable acidity level similar to commercial product, while maintaining acceptable syneresis within the observed range and enhanced firmness, a desirable attribute for yogurt‐like products. The selection of 55°C as a critical drying temperature is further supported by its dual role in promoting sufficient acidity and gel structure development, while avoiding excessive syneresis associated with higher temperatures. Although shorter fermentation durations (< 48 h) may preserve higher viscosity and limit retrogradation in FMS flour, they lead to insufficient acidification in the akpan. Conversely, excessively high drying temperature, while increasing setback and firmness, may negatively impact water retention and visual quality through increased syneresis. Therefore, a processing defined by approximately 60–72 h of fermentation and drying around 55°C can be considered optimal for producing FMS flour capable of yielding akpan with physicochemical and textural properties closely aligned with those of commercial akpan.

## 4 Conclusion

Fermentation duration and drying temperature significantly influenced the quality of fermented maize starch flour and the physicochemical and textural properties of the resulting akpan. Longer fermentation increased titratable acidity, whereas drying temperature affected acidity, setback, and syneresis. The pH of the fermented maize starch (FMS) flour and resulting akpan was comparatively less responsive to the processing conditions, while lactic acid content of the FMS flour remained relatively stable across the treatments. An optimal balance was identified at 60–72 h fermentation combined with a drying temperature of approximately 55°C, yielding shelf‐stable FMS flour and resulting in akpan with titratable acidity, syneresis, and firmness comparable to those commercial products. These findings provide a basis for standardizing FMS flour production and enhancing the scalability and market potential of akpan.

## Author Contributions

**Laurent Adinsi:** writing – original draft, formal analysis, conceptualization. **Agnes Mehoba:** data curation. **Fernande G. Honfo:** writing – review and editing.

## Funding

Authors greatly thank the “Programme de Productivité Agricole en Afrique de l'Ouest, PPAO” for financial supporting through the “Centre National de Spécialisation sur le Maïs du Bénin, CNS‐Maïs”. This work was supported by Programme de Productivité Agricole en Afrique de l'Ouest.

## Conflicts of Interest

The authors declare no conflicts of interest.

## Data Availability

The data that support the findings of this study are available from the corresponding author upon reasonable request.
